# A Comprehensive Review of Graft Choices and Surgical Techniques in Primary Anterior Cruciate Ligament Reconstruction: An Outcome Analysis

**DOI:** 10.7759/cureus.68701

**Published:** 2024-09-05

**Authors:** Milind R Gharpinde, Ankit M Jaiswal, Yash Dhanwani

**Affiliations:** 1 Orthopedics, Jawaharlal Nehru Medical College, Datta Meghe Institute of Higher Education and Research, Wardha, IND; 2 Orthopaedics, Jawaharlal Nehru Medical College, Datta Meghe Institute of Higher Education and Research, Wardha, IND

**Keywords:** acl reconstruction, anterior cruciate ligament, functional recovery, graft choices, knee stability, outcome analysis, surgical techniques

## Abstract

Anterior cruciate ligament (ACL) injuries are among the most prevalent knee injuries, particularly in athletes engaged in high-impact sports. ACL reconstruction is a widely performed surgical procedure to restore knee stability, prevent further knee damage, and enable patients to return to their previous physical activity levels. However, the success of ACL reconstruction is influenced by various factors, including the choice of graft and the surgical technique employed. This comprehensive review explores the outcomes of different graft options - autografts, allografts, and synthetic grafts - and various surgical techniques such as single-bundle versus double-bundle reconstruction and anatomic versus non-anatomic tunnel placement. The review analyzes the short- and long-term outcomes, including functional recovery, return to sports, complication rates, and the impact of patient-specific factors such as age, activity level, and comorbidities. Additionally, the review discusses the role of rehabilitation protocols in optimizing surgical outcomes. By synthesizing current evidence, this review aims to provide clinicians with insights into the most effective graft choices and surgical techniques for primary ACL reconstruction, ultimately guiding the optimization of patient outcomes and highlighting areas for future research.

## Introduction and background

The anterior cruciate ligament (ACL) is a critical structure in the knee joint that maintains stability during dynamic activities such as cutting, pivoting, and jumping [1]. ACL injuries are among the most common knee injuries, with an estimated 100,000 to 200,000 ACL ruptures occurring annually in the United States [2]. These injuries often result from non-contact mechanisms such as sudden deceleration, changes in direction, or awkward landings, though contact injuries are also possible. Such injuries are particularly prevalent in athletes participating in sports such as soccer, basketball, skiing, and football, where quick directional changes and high-impact movements are frequent [3]. An ACL rupture can significantly impair knee stability, severely limiting an individual’s ability to engage in sports or perform everyday activities. Without appropriate treatment, an ACL injury can lead to chronic knee instability, which increases the risk of further damage to the knee, including meniscal tears and early onset osteoarthritis. Therefore, ACL reconstruction is commonly recommended for active individuals who wish to return to their pre-injury level of physical activity or for those whose knee instability impacts their quality of life [1].

ACL reconstruction is a surgical procedure designed to restore knee stability and function by replacing the torn ligament with a graft. The primary goal of this surgery is not only to allow patients to return to their previous activity levels but also to prevent long-term complications such as recurrent instability, additional knee injuries, and the development of osteoarthritis [4]. Over the past few decades, ACL reconstruction has evolved into a routine procedure, thanks to advancements in surgical techniques and graft options. Despite its routine nature, the outcomes of ACL reconstruction can vary significantly, influenced by factors such as the type of graft used, the surgical technique employed, and the individual characteristics of the patient [5]. The choice of graft - autograft, allograft, or synthetic - can affect the healing process, graft integration, and long-term knee function. Additionally, variations in surgical techniques, such as single-bundle versus double-bundle reconstruction and anatomic versus non-anatomic tunnel placement, can have a substantial impact on the biomechanical stability of the knee and the overall success of the surgery [5].

This review aims to provide a comprehensive analysis of the outcomes associated with different graft choices and surgical techniques in primary ACL reconstruction. By examining the existing literature and comparing the efficacy, safety, and long-term results of various graft options and surgical methods, this review aims to guide clinicians in making informed decisions that optimize patient outcomes. This analysis seeks to identify best practices in ACL reconstruction and highlight areas where further research is needed to improve the success rates of this standard yet complex surgical procedure.

## Review

Anatomy and function of the ACL

**Figure 1:**
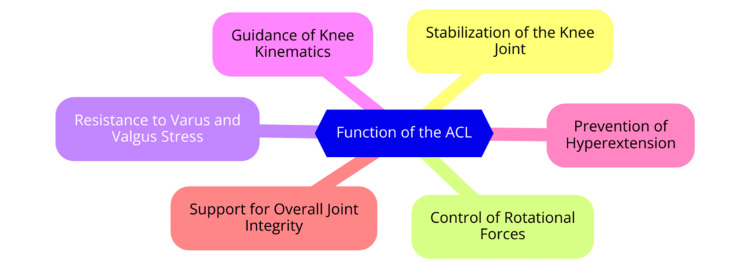
Functions of the ACL Image Credit: Dr Milind Gharpinde ACL, anterior cruciate ligament

The ACL is crucial in the knee joint and primarily stabilizes the knee against anterior tibial translation and rotational forces [6]. Structurally, the ACL is composed mainly of type I collagen, imparting strength and elasticity. It typically has an hourglass or bowtie shape, measuring between 27 to 38 mm in length and 10 to 12 mm in width [7]. The ligament is divided into two primary bundles: the anteromedial bundle (AMB) and the posterolateral bundle (PLB). These bundles serve distinct functions throughout the knee’s range of motion: the AMB tightens during knee flexion, providing stability against anterior translation, while the PLB becomes more engaged during knee extension, resisting rotational forces. This functional differentiation is vital for maintaining knee stability during various activities [8]. The ACL is essential for preserving knee stability by preventing excessive anterior translation of the tibia relative to the femur and controlling internal and external rotation. The ACL is estimated to provide around 85% of the total restraining force against anterior tibial translation. Additionally, it contributes to stabilizing the knee against varus and valgus stresses, thereby supporting overall joint integrity [9]. Along with the posterior cruciate ligament (PCL), the ACL helps guide the knee’s instantaneous center of rotation, critical for normal knee kinematics during movement. Proper ACL function is essential for athletic performance and everyday activities, as an ACL injury can lead to significant instability and an increased risk of further joint damage [10]. ACL injuries are among the most common knee injuries, particularly in sports. The majority of ACL injuries occur through non-contact mechanisms, often involving a combination of valgus stress and internal rotation of the knee. Activities involving sudden stops, direction changes, or awkward landings are hazardous. Injuries can also occur during contact situations, such as direct blows to the knee. ACL tears frequently occur with other injuries, such as damage to the medial collateral ligament (MCL) or menisci, which can further complicate treatment and recovery [11]. The incidence of ACL injuries is notably high among athletes, with an estimated occurrence of 78 to 84 per 100,000 individuals across various populations. Understanding the anatomy and function of the ACL is crucial for developing effective prevention strategies and treatment plans, especially in high-risk populations [12]. The functions of the ACL are illustrated in Figure 1.

Graft choices for ACL reconstruction

Selecting a graft for ACL reconstruction is a crucial decision that significantly influences surgical outcomes, recovery, and re-injury risk. The primary graft options for ACL reconstruction include autografts, allografts, and synthetic grafts [13]. Autografts derived from the patient’s tissue are widely favored due to their superior biological integration and biomechanical properties. The most commonly utilized autografts are the bone-patellar tendon-bone (BPTB), hamstring tendon (HT), and quadriceps tendon (QT) [14]. BPTB autografts offer robust bone-to-bone healing owing to the bone plugs at each end, facilitating quicker integration and stability. However, they are associated with anterior knee pain, extended recovery periods, and potential complications such as patellar fractures. HT autografts are preferred for minimizing donor site morbidity and providing biomechanical strength comparable to BPTB, typically resulting in less postoperative anterior knee pain [15]. Nonetheless, they may have slower healing times and a potential for muscle weakness in the harvested area. QT autografts combine the strengths of both BPTB and HT, offering a larger cross-sectional area that may enhance graft strength and stability. Despite these advantages, QT autografts are less commonly used, and there is limited long-term data compared to BPTB and HT [16]. Allografts, sourced from donor tissue, present an alternative to autografts, particularly for patients who wish to avoid the complications associated with donor site harvesting. Standard allografts include the tibialis anterior/posterior, Achilles tendon, and peroneus longus. Allografts can reduce surgical time and postoperative pain but may have slower incorporation and a higher risk of re-rupture than autografts [17]. Tibialis anterior/posterior allografts offer reduced donor site morbidity and quicker recovery but may exhibit slower integration and a higher risk of re-rupture. Achilles tendon allografts provide a strong graft option with favorable biomechanical properties; however, there is a risk of complications related to the harvesting site and the potential for slower integration. Peroneus longus allografts share similar benefits, including reduced recovery time, but they are less common and may yield variable outcomes [18].

Synthetic grafts, such as the Ligament Augmentation and Reconstruction System (LARS) ligament and Gore-Tex, offer a non-biological alternative for ACL reconstruction. LARS ligaments are designed to facilitate quick recovery with minimal donor site morbidity, but long-term outcomes are less favorable compared to biological grafts, with concerns regarding durability and integration. Gore-Tex grafts provide a synthetic option that circumvents biological graft complications, but they may not integrate well biologically, potentially leading to long-term complications [19]. Autografts generally exhibit superior initial strength and faster biological integration than allografts when evaluating the outcomes of different graft choices. However, allografts may be more convenient for certain patients due to reduced donor site morbidity [20]. Research indicates that autografts offer better knee stability and postoperative function, particularly in younger, more active patients. In contrast, allografts may be more appropriate for older individuals or those with lower activity levels. Autografts typically have lower re-rupture rates than allografts, especially in younger, athletic populations, with allografts being associated with higher rates of failure and reoperation, particularly in patients under 30 years of age [20]. Table 1 provides a comparison of graft choices for ACL reconstruction.

**Table 1:** Comparison of graft choices for ACL reconstruction ACL, anterior cruciate ligament; BPTB, bone-patellar tendon-bone; HT, hamstring tendon; QT, quadriceps tendon

Graft Type	Source	Advantages	Disadvantages/Considerations
Autograft	Patient's tissue (e.g., patellar tendon, hamstring, quadriceps tendon)	Excellent biological incorporation, lower risk of rejection, quicker healing	Potential donor site morbidity, longer recovery at the harvest site, risk of weakness in harvested area
BPTB autograft	Patient's patellar tendon with bone plugs from the patella and tibia	Intense bone-to-bone healing, quicker integration, and stable fixation	Associated with anterior knee pain, risk of patellar fracture, and potential for more prolonged recovery time
HT autograft	Patient's HTs (semitendinosus and/or gracilis)	Less anterior knee pain, lower donor site morbidity, and good biomechanical properties	Slower healing and potential for muscle weakness in the harvested area may result in lower graft stiffness
QT autograft	Patient's QT, with or without bone plug	A larger graft size, potentially stronger, combines the benefits of BPTB and HT	Less common, limited long-term data compared to BPTB and HT, possible donor site morbidity
Allograft	Donor tissue (e.g., tibialis anterior/posterior, Achilles tendon, peroneus longus)	No donor site morbidity, shorter surgery time, and reduced postoperative pain	Slower incorporation, higher re-rupture rates, potential for immune response, risk of disease transmission
Tibialis anterior/posterior allograft	Donor tibialis anterior or posterior tendon	Reduced donor site morbidity and quicker recovery are commonly used	Slower integration, higher re-rupture rates, and potential variability in graft quality
Achilles tendon allograft	Donor Achilles tendon	Strong graft option with good biomechanical properties	Risk of complications related to harvesting, slower integration, and larger graft may require more precise placement
Peroneus longus allograft	Donor peroneus longus tendon	Reduced recovery time and lower donor site morbidity	Less common, variability in outcomes, the potential for slower integration
Synthetic graft	Manufactured materials (e.g., LARS ligament, Gore-Tex)	No donor site morbidity, quick recovery, availability not dependent on donor tissue	Long-term outcomes are less favorable, with concerns over durability, biological integration, and potential for complications such as graft wear or failure

Surgical techniques in ACL reconstruction

Surgical techniques in ACL reconstruction have evolved significantly, with advancements in graft selection, tunnel placement, fixation methods, and minimally invasive approaches. These factors determine the procedure's success and the patient's overall recovery [21]. A primary distinction in ACL reconstruction is between single- and double-bundle techniques. Single-bundle reconstruction, the traditional approach, involves replacing the ACL with a single graft. In contrast, double-bundle reconstruction aims to replicate the ACL's native anatomy by reconstructing both the AMB and PLB [21]. Theoretically, double-bundle reconstruction offers advantages such as improved rotational stability and enhanced resistance to tibial translation, closely mimicking the natural ACL function. However, clinical outcomes have been mixed. Some studies suggest that double-bundle techniques result in lower graft failure rates and better stability outcomes [22]. Conversely, other research indicates that the differences in knee function and stability between single- and double-bundle reconstructions may not be as significant as initially believed, particularly in the short- to medium-term. This variability underscores the importance of considering individual patient factors when selecting the appropriate surgical technique [22]. The placement of tibial and femoral tunnels is another critical aspect of ACL reconstruction that can significantly impact outcomes. Anatomic tunnel placement, designed to replicate the original ACL attachment sites, enhances knee biomechanics and stability. Studies have shown that accurately placed tunnels restore normal knee kinematics, leading to better functional outcomes and lower graft failure rates [23]. In contrast, non-anatomic tunnel placements, which may not align with the native ACL's anatomy, can alter knee mechanics and increase the risk of complications. Research indicates that improper tunnel placement is associated with higher reoperation rates, emphasizing the importance of precise surgical techniques. Anatomic placement is, therefore, often favored to optimize the chances of a successful outcome [24].

The choice of fixation method during ACL reconstruction is another important consideration that can influence graft healing and clinical outcomes. Two commonly used fixation methods are interference screws and cortical buttons. Interference screws are widely adopted for their ease of application and ability to provide strong fixation, allowing for immediate stability [25]. However, cortical buttons, often used in double-bundle reconstructions, can offer advantages in certain anatomical configurations by providing better fixation and potentially reducing the risk of tunnel widening. Choosing between these fixation methods can impact the graft's mechanical properties and integration into the host tissue. Some studies suggest that cortical button fixation may improve graft healing and lower failure rates, particularly in specific contexts, highlighting the need for tailored approaches based on individual patient anatomy and surgical goals [26]. Finally, the trend toward minimally invasive surgical techniques has transformed ACL reconstruction. Arthroscopic surgery is now the standard of care, offering numerous advantages over traditional open surgical approaches. The minimally invasive nature of arthroscopy results in less postoperative pain, reduced scarring, and quicker recovery times. Additionally, arthroscopic techniques allow for better visualization and precision in graft placement, which is crucial for successful outcomes. Patients who undergo arthroscopic ACL reconstruction typically experience faster rehabilitation and a quicker return to their pre-injury activity levels compared to those who undergo open surgery. Studies consistently show that arthroscopic techniques lead to lower complication rates and better overall functional outcomes, reinforcing the benefits of this approach in modern ACL reconstruction [27]. Table 2 provides an overview of surgical techniques in ACL reconstruction.

**Table 2:** Overview of surgical techniques in ACL reconstruction ACL, anterior cruciate ligament

Surgical Technique	Description	Advantages	Disadvantages/Considerations
Single-bundle reconstruction	It uses a single graft to replace the ACL, which is traditionally the standard approach	Simpler procedure, shorter operative time, proven long-term outcomes	It may provide less rotational stability compared to double-bundle techniques
Double-bundle reconstruction	Reconstructs the anteromedial and posterolateral bundles of the ACL to replicate native anatomy	Improved rotational stability and better resistance to tibial translation	More complex surgery, longer operative time, and mixed clinical outcomes in comparison to single-bundle technique
Anatomic tunnel placement	Tunnels are placed to closely replicate the original ACL attachment sites	Restores normal knee kinematics, better functional outcomes, and lower graft failure rates	Requires precise surgical technique, and improper placement can lead to poor outcomes
Non-anatomic tunnel placement	Tunnels are placed without strict adherence to the original ACL attachment sites	Easier to perform, potentially less surgical time required	Altered knee mechanics, increased risk of complications, and higher reoperation rates
Interference screw fixation	Fixes the graft using screws placed within the bone tunnels	Strong initial fixation allows for immediate stability and is commonly used and well-studied	Potential for graft laceration, tunnel widening, and complications related to screw placement
Cortical button fixation	Uses a button on the cortical surface of the bone for graft fixation, often used in double-bundle technique	Strong fixation, particularly in double-bundle reconstructions, less risk of tunnel widening	It is more technically demanding and may not be suitable for all anatomical configurations
Arthroscopic techniques	A minimally invasive approach using arthroscopy for graft placement and fixation	Reduced postoperative pain, quicker recovery, lower complication rates, better visualization during surgery	It requires specialized equipment and training, has higher costs, and has potential technical challenges

Outcome analysis

The outcome analysis of primary ACL reconstruction involves evaluating various factors, including short-term and long-term outcomes, functional recovery, complications, and patient-specific influences [28]. Short-term outcomes typically focus on recovery within the first year post-surgery, with research indicating that various surgical techniques yield comparable subjective outcomes, such as those measured by the International Knee Documentation Committee (IKDC) and ACL Return to Sport after Injury (ACL-RSI) scores. For example, a recent study found no significant differences in subjective knee stability and pain levels across different ACL reconstruction techniques during short-term follow-ups (ranging from six weeks to nine months) [28]. Long-term outcomes often examine the durability of the graft and the incidence of complications, such as graft failure and osteoarthritis. Research has shown that patients remain at a higher risk of subsequent ACL injuries within the first two years following reconstruction compared to those without a prior injury. Long-term studies also suggest that the type of graft used can influence outcomes, with certain grafts demonstrating better longevity and lower re-rupture rates [29]. The ability to return to pre-injury sports levels is a key indicator of success following ACL reconstruction. Many studies report that approximately 60-80% of athletes return to their previous activity levels, although this can vary significantly based on age, sex, and the type of graft used. Younger patients tend to have higher return rates, while older patients may face more challenges in returning to their pre-injury activity levels [30]. Patient-reported outcome measures are essential for assessing the subjective success of ACL reconstruction. Tools such as the IKDC and ACL-RSI provide valuable insights into patients' perceptions of knee function and overall satisfaction with the surgery. Short-term studies generally report good-to-excellent outcomes, with many patients experiencing significant improvements in knee function and quality of life [31].

Infection rates following ACL reconstruction are generally low but remain a critical concern. Complications such as infections can lead to increased morbidity and the need for additional surgical interventions. Graft failure and re-rupture rates are also significant considerations in outcome analyses. Studies indicate that these rates can vary based on the graft type, with younger patients exhibiting higher risks of re-injury [32]. Overall, re-rupture rates range from 5% to 20%, depending on various factors, including surgical technique and adherence to rehabilitation protocols. The need for secondary surgeries, such as revisions or additional procedures to address complications, is another important metric. Rates of secondary surgeries can be influenced by the initial surgical technique and patient factors, with some studies reporting rates as high as 10-15% within the first few years post-reconstruction [33]. Patient demographics play a crucial role in outcomes. Younger athletes generally achieve better outcomes and higher return-to-sport rates than older individuals. Additionally, male patients often report higher activity levels and lower rates of complications. Pre-existing conditions, such as meniscal injuries or osteoarthritis, can negatively impact surgical outcomes. Patients with a history of knee injuries may face additional challenges in recovery and returning to activity. Studies suggest addressing these conditions before ACL reconstruction can improve overall outcomes [34].

Rehabilitation protocols

Following ACL reconstruction, rehabilitation is crucial for optimal recovery and a successful return to sports. This process is typically organized into phased rehabilitation programs, each with specific goals and criteria for progression. The approach taken during rehabilitation significantly influences surgical outcomes, including graft healing and functional recovery [35]. Rehabilitation is generally divided into several phases, each targeting different objectives. In the initial phase (zero to two weeks post-surgery), the primary goals are to protect the graft, reduce swelling, restore patellar mobility, and achieve full knee extension. Patients engage in gentle range-of-motion exercises, patellar mobilization, and quadriceps strengthening, all while using crutches and maintaining a straight knee at rest [36]. The second phase (two to six weeks post-surgery) continues to protect the graft while improving the range of motion and beginning strengthening exercises. This phase includes gradually introducing weight-bearing activities and more intensive exercises, such as leg presses and hamstring curls. Proprioception training is also introduced during this stage [37]. The third phase (six to 12 weeks post-surgery) aims to maintain a full range of motion and safely advance strengthening exercises, incorporating more complex activities such as single-leg movements and plyometrics. Patients are assessed for stability and strength during this phase before progressing further. The final phase (three to six months post-surgery) is focused on sport-specific training and maintaining overall strength. This phase emphasizes sport-specific drills and a gradual return to athletic activities, with ongoing assessments of functional capabilities [38]. The decision to return to sports is based on the time elapsed since surgery and functional criteria. These criteria may include achieving full range of motion in comparison to the uninjured leg, demonstrating sufficient strength (e.g., quadriceps strength greater than 90% of the uninjured leg), and successfully passing functional tests such as single-leg squats and agility drills without experiencing pain or instability [39]. Rehabilitation is critical for graft healing. Early restoration of range of motion is essential to prevent stiffness and promote proper graft healing. Studies indicate that starting rehabilitation promptly can enhance graft integration and reduce the risk of complications, such as joint stiffness and muscle atrophy. Furthermore, effective rehabilitation is associated with improved functional recovery and lower re-injury rates. A well-structured rehabilitation program restores strength and mobility and mentally prepares the athlete for a safe return to sports. Research suggests that athletes who adhere to comprehensive rehabilitation protocols are less likely to experience re-injury compared to those who do not follow structured rehabilitation plans [40].

Emerging trends and future directions

Emerging trends and future directions in ACL reconstruction are centered on advancements in biological augmentation, innovations in surgical techniques, and the need for comprehensive long-term outcome studies. Biological augmentation methods, such as platelet-rich plasma and stem cells, are increasingly recognized as promising options for enhancing ACL repair and reconstruction. These biological agents aim to create a more conducive healing environment for the graft, which could lead to improved clinical outcomes. Early research suggests that incorporating biological agents into ACL reconstruction can positively affect graft healing and integration, potentially reducing side-to-side differences and failure rates [41]. However, these methods are still in the exploratory phase, and more clinical evidence is needed to confirm their efficacy and safety for widespread use. The significance of biological augmentation lies in its potential to boost the intrinsic healing capacity of the ACL, which is naturally limited due to its hypovascular nature. Studies indicate that biological therapies can enhance the biomechanical properties of the graft and promote better integration with surrounding tissues [42].

Innovations in surgical techniques are also driving the future of ACL reconstruction. Robot-assisted surgery is an emerging advancement that offers enhanced precision in graft placement and tunnel drilling. This technology seeks to improve surgical outcomes by standardizing procedures and reducing the variability introduced by human factors during surgery. Moreover, the development of 3D printing technology is revolutionizing ACL reconstruction by enabling the creation of personalized grafts tailored to each patient's anatomy. These customized grafts are expected to improve graft fit and integration, thereby increasing success rates and minimizing the risk of complications associated with standard graft sizes and shapes [43]. Long-term outcome studies are essential for evaluating the durability of surgical interventions and the sustained functionality of the ACL over time. There is a growing recognition of the need for standardized outcome measures in ACL reconstruction research. Consistent metrics will allow for better comparison across studies and enhance our understanding of long-term outcomes related to graft types and surgical techniques. Additionally, many current studies lack adequate follow-up periods, which can obscure the true success rates and potential complications of different treatment modalities. Future research should emphasize extended follow-up to collect comprehensive data on patient outcomes over the long term [44]. Table 3 illustrates the emerging trends and future directions in ACL reconstruction.

**Table 3:** Emerging trends and future directions in ACL reconstruction ACL, anterior cruciate ligament

Trend/Direction	Description	Potential Benefits	Challenges/Considerations
Biological augmentation	Use of platelet-rich plasma and stem cells to enhance graft healing and integration	Improved graft healing, better clinical outcomes, and reduced failure rates	It is still in exploratory stages and requires further clinical evidence for widespread adoption
Robot-assisted surgery	Utilization of robotic technology for enhanced precision in graft placement and tunnel drilling	Increased surgical precision, reduced complications, and standardized procedures	High cost, need for specialized training, and limited availability in some regions
3D printing technology	Creation of personalized grafts tailored to individual patient anatomy	Improved graft fit, enhanced integration, and potentially better overall outcomes	Need for further research to determine long-term success; higher initial costs
Long-term outcome studies	Focus on extended follow-up periods to assess the durability and functionality of ACL reconstructions	Comprehensive data on long-term patient outcomes, a better understanding of graft longevity	It requires significant time and resources; there is a need for standardized outcome measures across studies
Standardization of outcome measures	Establishing consistent metrics for evaluating ACL reconstruction outcomes across studies	Facilitates better comparisons and improves understanding of long-term success rates	Agreement on standardized measures may be challenging; implementation across studies requires coordination
Enhanced rehabilitation protocols	Continued evolution of rehabilitation strategies to optimize post-operative recovery and return to sport	Faster recovery, improved functional outcomes, reduced risk of re-injury	Individual variability in response to protocols and adherence to rehabilitation may vary among patients

## Conclusions

ACL reconstruction remains a critical procedure for restoring knee stability and function in individuals with ACL injuries, particularly those aiming to return to high physical activity levels. The choice of graft and surgical technique plays a pivotal role in determining the success of the surgery, influencing both short-term recovery and long-term outcomes. Autografts, allografts, and synthetic grafts each offer distinct advantages and challenges. At the same time, surgical techniques such as single-bundle versus double-bundle reconstruction and anatomic versus non-anatomic tunnel placement can significantly impact knee biomechanics and stability. Through a comprehensive analysis of current literature, this review underscores the importance of personalized surgical approaches tailored to each patient's specific needs. While considerable progress has been made in optimizing ACL reconstruction, ongoing research is essential to refine graft selection, enhance surgical techniques, and improve patient outcomes. As the field continues to evolve, integrating emerging technologies and innovations promises to advance the success and longevity of ACL reconstruction procedures.
